# Cannabis industry lobbying in the Colorado state legislature in fiscal years 2010–2021

**DOI:** 10.1016/j.drugpo.2022.103585

**Published:** 2022-01-24

**Authors:** Thomas Rotering, Dorie E Apollonio

**Affiliations:** Department of Clinical Pharmacy, University of California, UCSF Clinical Sciences Box 0622, 521 Parnassus Avenue, Floor 3 Room 3303, San Francisco, CA 94143, USA

**Keywords:** Cannabis, Lobbying, Policy, Public health, Colorado

## Abstract

**Background:**

The cannabis industry has an interest in creating a regulatory environment which maximizes profits at the cost of public health, similar to the tobacco, alcohol, and food industries. This study sought to describe the cannabis industry’s lobbying activities in the Colorado State Legislature over time.

**Methods:**

This retrospective observational study analyzed publicly available lobbying expenditures data from fiscal years (FY) 2010–2021. Measures included inflation-adjusted monthly lobbying expenditures by funder and lobbyist, origin of funding, and lobbyist descriptions of cannabis industry clients. This dataset was supplemented with business license documentation, legislative histories, and public testimony.

**Results:**

The cannabis industry spent over $7 million (inflation adjusted) from FY 2010–2021 to lobby the Colorado legislature on 367 bills. Over $800,000 (11% of total cannabis spending) was from out-of-state clients. In 48% of lobbyist reports lobbyists did not disclose their funder’s cannabis affiliation, and cannabis organizations used strategies that may have obscured the true amount and source of funding. Lobbyists and agencies concurrently represented the alcohol, tobacco, and cannabis industries, possibly facilitating inter-industry alliances when interests align.

**Conclusion:**

The cannabis industry dedicated significant resources towards lobbying the Colorado State Legislature on behalf of policies intended to increase cannabis use. Creating transparency about the relationships between the cannabis industry, related industries, and policymakers is essential to ensure appropriate regulation of cannabis products.

## Introduction

Medical cannabis use was illegal throughout the US until 1996, and recreational use was illegal until 2012. As of August 2021, 18 US states, the District of Columbia, Guam, and the Northern Marianas Islands had passed laws permitting recreational and medical cannabis and 17 states permitted only medical cannabis (Weiser, 2021). Supporters’ reasoning for legalization includes arguments about therapeutic benefits, redirecting law enforcement to violent crimes, personal freedom, tax revenues, product regulations, and harmlessness (Jones, 2019). Both recreational and medical legalization increase cannabis use (Cerdá et al., 2020). In Colorado, the first state to legalize adult-use cannabis in 2012, past 30-day cannabis use increased among those aged 18–25 from 26.8% in 2011 to 34.4% in 2018 (Substance Abuse & Mental Health Services Administration [SAMHSA], 2021). The regulated cannabis market in Colorado registered $10 billion in sales between 2014, when adult-use sales began, and 2020, when sales reached $2.19 billion (Colorado Department of State [CDOS], 2021b).

Cannabis smoking, overwhelmingly the most common form of cannabis consumption (Dai & Richter, 2019), exposes users to many of the same toxins contained in tobacco smoke, including particulate matter (PM2.5), polycyclic aromatic hydrocarbons, gasses, and volatile organic compounds (Moir et al., 2008). Cannabis use is associated with more frequent chronic bronchitis episodes, airway injury, myocardial infarction, and ischemic stroke (National Academies of Sciences, Engineering, and Medicine [NASEM], 2017; Rumalla, Reddy & Mittal, 2016; Shah, Patel, Paulraj & Chaudhuri, 2020). Secondhand cannabis smoke also poses a risk to nonsmokers (Glantz, Halpern-Felsher & Springer, 2018; Herrmann et al., 2015; Murphy, Huang & Schick, 2021; Wilson et al., 2018).

Commercial determinants of health research, which studies the commercial drivers of poor health outcomes, has identified mechanisms of influence that the tobacco, food, and alcohol industries employ to promote products in ways that compromise public health (Kickbusch, Allen & Franz, 2016). Tobacco, alcohol, and gambling companies, for example, hire lobbyists to influence policy, connect with front groups and allied industries to oppose regulation, and build relationships with policymakers through political donations (Kypri et al., 2019). Tobacco, (Saloojee & Dagli, 2000) alcohol, (Babor, Robaina, & Noel, 2018; McCambridge, Mialon & Hawkins, 2018; Miller & Harkins, 2010) and food (Miller & Harkins, 2010) interests orchestrate lobbying across industries and transnationally to promote policies favorable to consumption. The cannabis industry has a similar interest in maximizing profits by creating a favorable regulatory environment.

Cannabis corporations share links with the alcohol and tobacco industries. Tobacco companies Altria (Roberts, 2021), Imperial Brands (Auxly Cannabis Group, Inc., 2019), and British American Tobacco (BAT, 2021), have all made significant investments in cannabis, a long-anticipated development (Barry, Hiilamo & Glantz, 2014). Constellation Brands, maker of Corona beer, has also made investments in Canopy Growth, a Canadian cannabis corporation (Nair, 2020). Tobacco and alcohol interests have openly formalized a cannabis-focused political association as members of the Coalition for Cannabis Policy, Education, and Regulation, a lobbying group that lists Altria, Constellation Brands, and Molson Coors Beverage Company as members (CPEAR, 2021). Employing tactics used by the tobacco industry for decades (Crompton, 1993), cannabis companies are also vested in major sports through sponsorship of athletes and leagues in the US (Wise, 2021).

Considering the health risks involved with cannabis use and the conflict between public health and the commercial interests of these industries, systematic analyses of cannabis industry influence on policymaking are essential. There has been little study on the topic despite several calls for research (Adams, Rychert & Wilkins, 2021; Hall & Lynskey, 2016; Shover & Humphreys, 2019). Although there have been popular media reports on cannabis industry lobbying expenditures, (Bunch, 2014; Fish, 2019; Ingold, 2013; Olinger, 2018) we identified no systematic analyses that assessed cannabis lobbying over time or identified connections between the cannabis industry and affiliates. Cannabis products are legal in multiple states, while remaining illegal (except for hemp products) at the federal level. Even though federal law technically supersedes state law, gaps in enforcement have been carved out by the federal government to allow for state legalization of adult-use and medical cannabis (Chemerinsky, Forman, Hopper & Kamin, 2015). As a result, it remains to be seen whether cannabis industry efforts to influence policy are comparable to other industries for which recreational consumption has historically been legal.

In this study we sought to describe cannabis industry lobbying in the Colorado state legislature, which dictates product standards, licensing requirements, and other policies relevant to cannabis sales. We hypothesized that the cannabis industry would use strategies similar to those of other similar industries including relying on hired lobbyists (Saloojee & Dagli, 2000), obscuring industry funding, (Apollonio & Bero, 2007) working with related industries, (Nguyen, Glantz, Palmer & Schmidt, 2019) and building national networks to support policies likely to increase consumption (Fallin, Grana & Glantz, 2014; Kalra, Bansal, Wilson & Lasseter, 2017). We focused on Colorado because it was the first state to legalize recreational cannabis in 2012, making it possible to assess whether cannabis industry lobbying activities have become comparable to other industries in nature and scope over time. Because of the complexity of relationships between the cannabis industry, lobbyists, and government officials, we supplemented the quantitative analyses with a case study illustrating cannabis industry tactics to influence the Colorado legislature.

## Methods

This retrospective observational study combined public lobbying data, business information, and legislative histories to describe cannabis industry lobbying in the Colorado state legislature between Fiscal Year 2010–2021.

### Setting and data

Colorado requires lobbyists to file reports on their activities with the Secretary of State, even if they are a salaried employee of the business they represent (CDOS, 2021a; Colorado Sunshine Act, C.R.S. § 24–6-301(1.9)(XI) (1962 & rev. (2020)).). From February to September 2021, we collected data on lobbying expenditures originating from the cannabis industry and its affiliates, from July 1, 2009 (beginning of the 2010 fiscal year before the second regular legislative session of the 67th General Assembly) to June 30, 2021 (the end of the 2021 fiscal year after adjournment of the first regular session of the 73rd General Assembly). The Colorado Department of State (CDOS) dataset details payments to registered lobbyists, with information on funders who hire lobbyists (referred to as “clients”), bill/rule titles and positions (Supporting, Amending, Opposing, or Monitoring) associated with payments, and lobbyist identifying information (CDOS, 2016).

To identify cannabis industry affiliates, we reviewed all funders in this dataset that lobbied on a list of 453 bills in fiscal years 2010–2021 that included the words “cannabis,” “marijuana,” or “hemp”. Using the CDOS business database, the Colorado Marijuana Enforcement Division search tool, and internet searches, we coded funders as cannabis affiliates if they a) held a cannabis business license, b) shared board members, owners, or investors with a cannabis company, c) disclosed members that were cannabis businesses, or d) would directly profit from cannabis sector growth (e.g., pharmaceutical companies that sell cannabis derived drugs, cannabis focused consultants, investors, lab services, or employee training services, etc.). For each lobbyist employed by a cannabis affiliate we examined their other funders and identified additional cannabis affiliates using the same inclusion criteria.

Because the CDOS dataset does not include lobbying payments made without a connection to a specific bill, administrative rule, or issue, we expanded the dataset by manually appending payments from cannabis affiliates in months where no lobbying was conducted for a specific bill/rule. Including these “retainer” payments allowed more accurate assessment of lobbying expenditures, because some funders make monthly payments to lobbyists rather than hiring them on an ad hoc basis. Funders also make payments to lobbyists before and after legislative sessions for work during the session. The completed search yielded a list of 1703 monthly payments from 89 cannabis affiliates with linked information on lobbyists they employed, positions on bills, and addresses.

Each lobbying report available on the CDOS website included an “industry type” field where lobbyists provide a description of the funder’s business. We coded these disclosures as “transparent” if the name or description contained a reference to cannabis, marijuana, or hemp and “ambiguous” if it did not.

Cannabis industry affiliates could be represented by lobbying agencies, lobbyists, and subcontractors. Cannabis affiliates may pay individual lobbyists or pay lobbying agencies (e.g., Gold Dome Access) that funnel those payments to salaried lobbyists or subcontractors. Lobbying agencies sometimes list themselves as funders even though this practice was made illegal by the Lobbyist Transparency Act, 2019. We excluded reported self-funding because it was impossible to identify the underlying funder. To prevent double counting, we only included direct payments from cannabis affiliates and excluded payments to subcontractors and employees salaried by lobbying agencies.

### Measures

Our primary measure was lobbying expenditures, which we adjusted for inflation using consumer price index (CPI) data from the U.S. Bureau of Labor and Statistics (Bureau of Labor & Statistics, 2021). We coded for cannabis affiliation, date of payments, address of funders, names and addresses of lobbyists, self-reported industry type, industry type identified through business records, and positions on proposed legislation.

### Analytical strategy

We reviewed cannabis lobbying expenditures in Colorado over time using Stata 16 and then qualitatively reviewed lobbying positions on proposed legislation. Our analyses assessed (a) total cannabis lobbying expenditures and the share drawn from national (out-of-state) sources, (b) the extent to which expenditures were clearly identified as associated with cannabis, and (c) alliances with other industries. We conclude with a case study of cannabis industry efforts to create cannabis consumption establishments. We selected this issue because legislation on the topic was introduced multiple times over the course of three years and under two gubernatorial administrations, allowing insight into changes in lobbying practices over time. We collected data from audio recordings of legislative testimony and floor debate, legislative histories, fiscal notes, and lobbying reports for all legislation dealing with cannabis consumption establishments available through the Colorado General Assembly and Secretary of State websites. We present a narrative description of each bill’s legislative history, including information from lobbying reports and demonstrative quotations made in public testimony that indicate cannabis industry influence in the policymaking process.

## Results

### Extent of cannabis industry spending

**Fig. 1.**
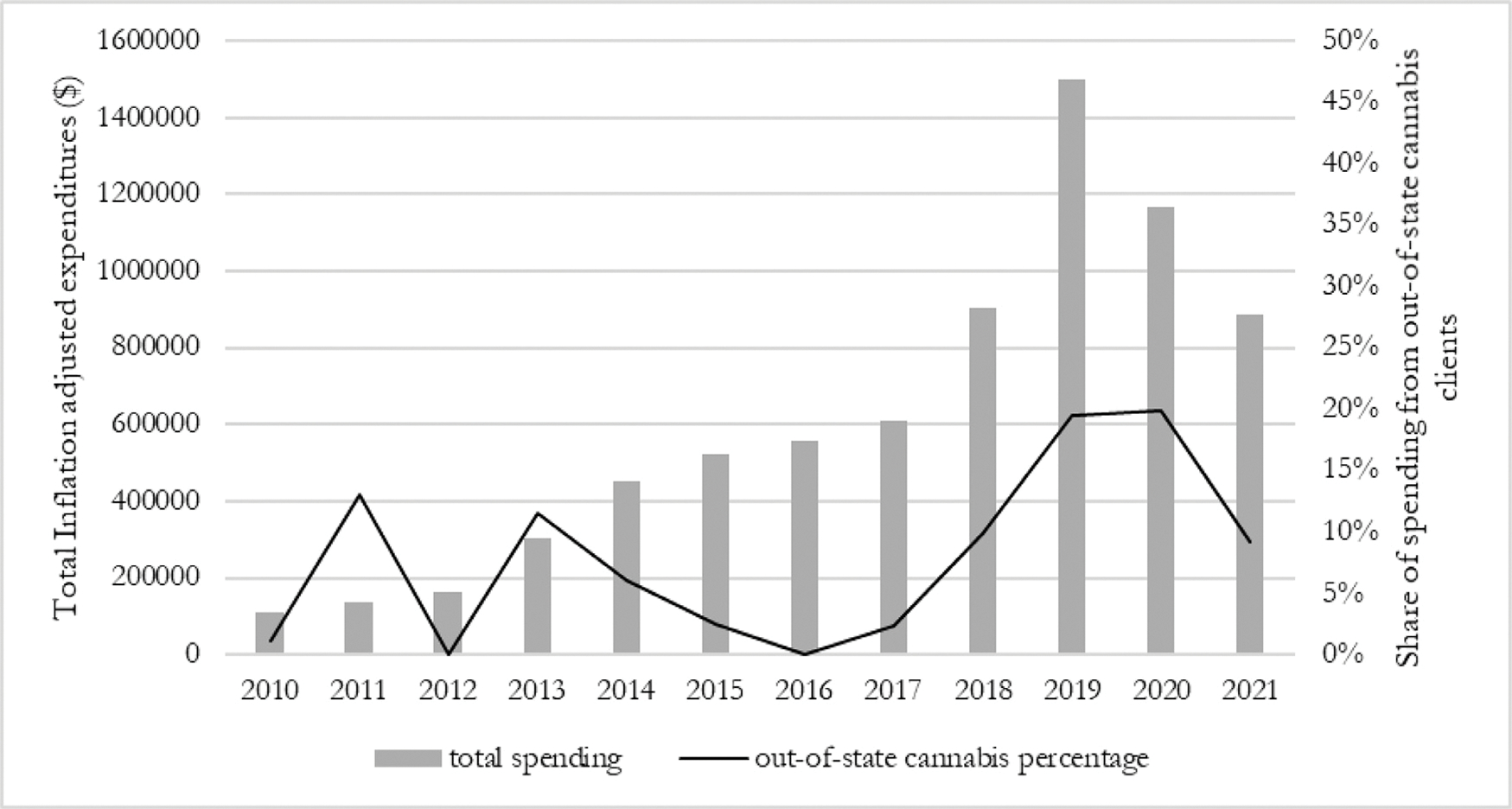
Total lobbying expenditures by the cannabis industry and share of out-of-state spending by fiscal year.

**Fig. 2.**
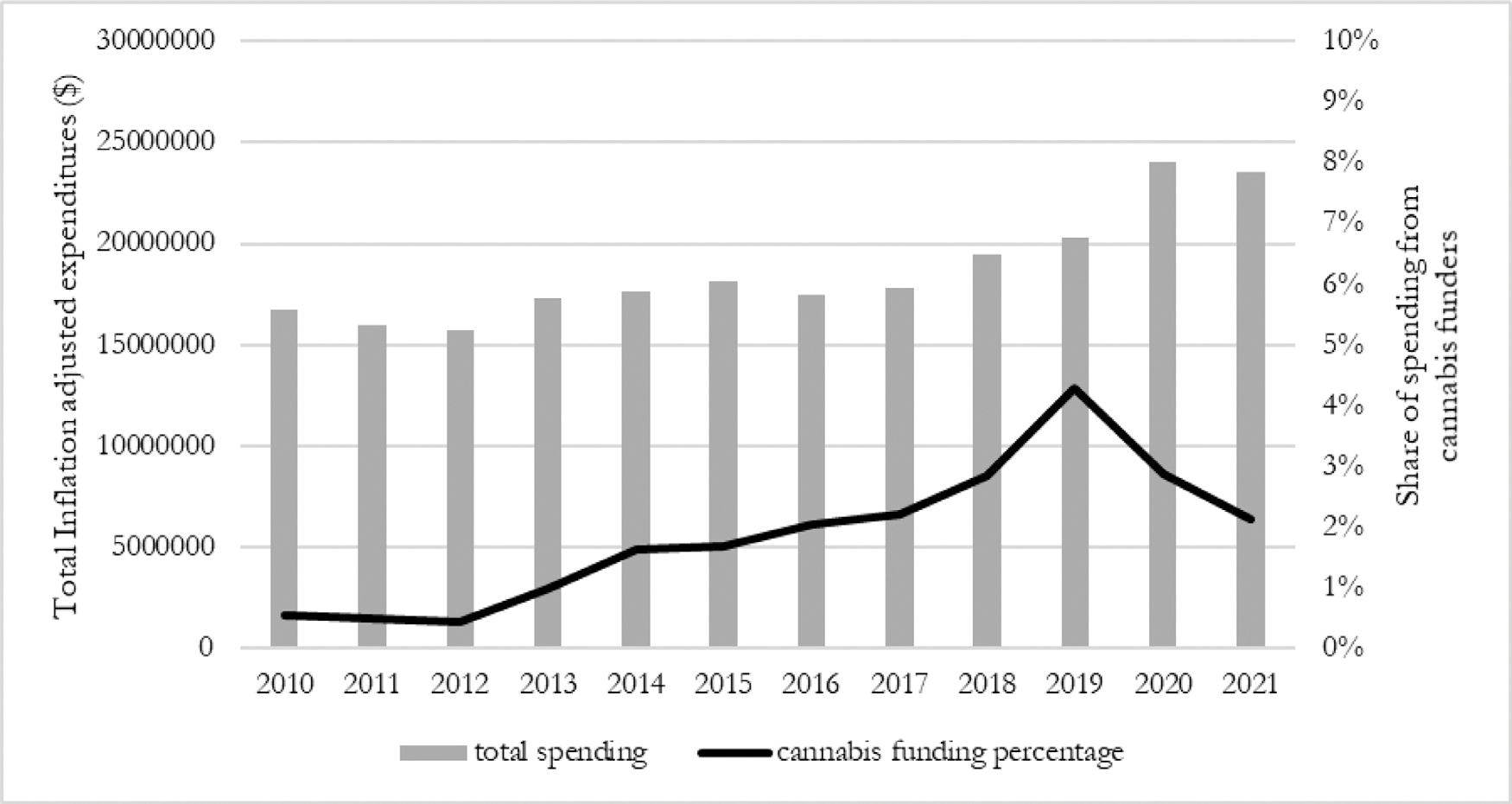
Total lobbying expenditures (excluding all retainer payments) from all sources and share of spending from cannabis industry funders by fiscal year.

Between fiscal years 2010 and 2021, 89 cannabis industry affiliates spent $7,345,585 lobbying the Colorado state legislature. After legalization in November 2012, annual lobbying expenditures increased by over 13 times, from $108,725 in fiscal year 2010 to a peak of $1,498,096 in fiscal year 2019 (Fig. 1). Lobbying expenditures from all sources (excluding retainer payments) grew at a slower rate, from $16,671,768 in 2010 to $19,667,714 in 2019. The share of spending attributable to cannabis interests increased relative to overall lobbying expenditures, from 0.54% in 2010 to 4.29% in 2019 (Fig. 2). The number of apparent cannabis funders increased from 7 in 2010 to 37 in 2019.

Many cannabis affiliates that appeared independent shared professional or personal ties. In 2019, 14 different funders lobbied in support of HB1090, a bill that allowed publicly traded corporations to own or invest in cannabis businesses and removed residency requirements. These 14 funders were exclusively cannabis affiliates or lobbying agencies with known cannabis industry connections: LivWell, Buddy Boy, Dixie Brands, Gobi Labs, Gold Dome Access, Lightshade, Medicine Man, MedPharm Holdings, Native Roots, Natural Selections, TEQ Analytic Solutions, The Green Solution, Vicente Sederberg, and Wolf Public Affairs. All but Gobi Labs shared professional ties: John Fritzel was an owner of both Lightshade and Buddy Boy, (Hubbard, 2018) and Andy Williams was the president of both Medicine Man and MedPharm Holdings (Andy Williams, 2021). Representatives from Lightshade, LivWell, Native Roots, Vicente Sederberg, Medicine Man, MedPharm Buddy Boy, Dixie Brands, and Columbia Care (which purchased The Green Solution following the bill’s passage) were board members or donors for the Cannabis Trade Federation. Leadership from Medicine Man, MedPharm Holdings, Native Roots, Dixie Brands, TEQ Analytical Solutions, Vicente Sederberg and the chairman of the Marijuana Industry Group all sat on the Board of Directors for Colorado Leads, an alliance of cannabis businesses. Lobbying records also indicated that Gold Dome Access represented the Marijuana Industry Group, Wolf Public Affairs represented Vicente Sederberg, and David Nagel lobbied for both TEQ Analytical Solutions and Natural Selections. Cannabis clients often shared the same lobbyists/agencies (Table 1).

Cannabis industry affiliates paid lobbyists to monitor amend, support, or oppose 367 bills between fiscal years 2010–2021. Of these bills, 220 (60%) mentioned the words cannabis, marijuana, or hemp, and dealt with issues related to licensing and physical requirements for cannabis businesses, biomedical research, public safety, product standards, and public education. Examples include support for HB16–1373, which allowed primary caregivers to administer medical cannabis to K-12 public school students and opposition of HB15–1298, which would have prohibited cannabis retailers from advertising to pregnant women and required signage warning pregnant women about the potential risks caused by cannabis use.

### Origins of Colorado cannabis funding

Cannabis industry affiliates with an out-of-state address spent $802,983 between fiscal years 2010–2021 (11% of cannabis spending). Given that some cannabis businesses are multistate operations with locations in Colorado and others use in-state PO boxes, this proportion is likely an underestimate. Immediately following adult-use legalization in November 2012 and prior to the creation of the recreational sales market in January 2014, the Washington D.C. based nonprofit Marijuana Policy Project dramatically increased its expenditures in Colorado. The proportion of out-of-state lobbying expenditures increased from 5.5% of lobbying expenditures in fiscal years 2010–2015 to 12.6% in fiscal years 2016–2021 (Fig. 2). California-based cannabis organizations lobbying in Colorado increased from one business spending $14,492 in 2017 to five spending $153,220 in 2020. One cannabis affiliated organization each from Ontario (Canopy Growth Corporation), New York (Nuka Enterprises), and Oregon (ORCL) lobbied in Colorado, as well as two from Washington D.C. (Marijuana Policy Project and UFCW Local 7).

### Transparency

In 48% of cannabis industry lobbying reports representing $3,147,491 (43% of expenditures), lobbyists used an ambiguous description of their funders’ affiliations. Lobbyist descriptions of funders are discretionary (Colorado Sunshine Act, C.R.S. § 24–6-301(1.9)(XI) (1962 & rev. (2020)).). Although some lobbyists described their funders as “Marijuana Dispensaries,” “Cannabis Industry,” or “Marijuana,” others used characterizations such as “Medical,” “General Business,” or “Food Services.” Ambiguous identifications also included acronyms like “CBA,” “NSG,” or “ORCL” or tradenames that required cross-referencing of addresses, licenses, and names to identify. Some lobbyists used the names of individuals who owned cannabis businesses rather than the business name.

Although we did not include it in our count of cannabis lobbying expenditures, funding from cannabis-affiliated public relations agencies was another potential source. Nine public relations agencies with known cannabis industry connections disbursed $1,051,898 to lobbyists working on 355 bills in the study period; 71/355 (20%) contained the keywords “cannabis,” “marijuana,” or “hemp”. Sewald Hanfling Public Affairs reported the largest amount of funding with $839,327 distributed to salaried employees working on cannabis-related bills. Sewald Hanfling was also itself paid at least $587,495 by cannabis businesses directly. Public relations agency salaries may reflect cannabis funding passed on to their employees; alternatively, these agencies may act as front groups for funders seeking to remain anonymous. The latter is suggested by Sewald Hanfling lobbyists listing an agency salary as their only source of income but also including the positions and identities of the cannabis businesses paying for representation, even if they were not listed as Sewald Hanfling funders.

### Inter-industry alliances

HB1076 (2019) removed exemptions to clean indoor air policies and added e-cigarette use to the definition of smoking and was initially opposed by tobacco interests including Reynolds American and the International Premium Cigar and Pipe Association, as well as the cannabis affiliate Renaissance Solutions. Renaissance Solutions changed its position from opposing to monitoring the bill two days after the passage of an amendment that exempted cannabis retailers from the Colorado clean indoor air act. Altria Client Services (an affiliate of Phillip Morris), Smoker Friendly, the Cannabis Business Alliance, the Colorado Gaming Association, the Colorado Petroleum Marketers Association, and the Medical Marijuana Industry Association had all sought to amend the bill.

Lobbyists employed by cannabis affiliates represented both that industry and other industries. Although some lobbyists exclusively represented cannabis affiliates (Jordan Welington, Kyle Forti, Nico Pento, Joe Megyesy, Sarah Woodson, Kevin Gallagher, Cherish St. Denis, Peter Marcus, Tyler Henson, Christian Sederberg, and lobbying agencies Vicente Sederberg, IComply and Tetra Public Affairs), others lobbied for cannabis and the tobacco, alcohol, pharmaceutical, and gaming industries. This shared representation may have allowed opportunities for inter-industry alliances. Axiom Strategies represented cannabis affiliates including the Medical Marijuana Industry Group, the Colorado Cannabis Chamber of Commerce, and Folium Biosciences in addition to the International Premium Cigar and Pipe Association, Altria Client Services, Reynolds American, Wine and Spirits Wholesalers of Colorado, Alkermes (a biopharmaceutical corporation) and Isle of Capri Casinos. Axiom Strategies’ largest client was HCA Healthcare. Margaret-Mary “Peggi” O’Keefe concurrently represented the Cannabis Business Alliance, the Colorado Cannabis Manufacturers Association, Altria Client Services, The Colorado Gaming Association, the Generic Pharmaceutical Association, and Mylan (a pharmaceutical company). Capitol Focus, LLC represented Gold Dome Access, the Marijuana Industry Group, the Colorado Gaming Association, Genetech, Glaxosmithkline, Johnson and Johnson, The Wine Institute, The Wine and Spirit Wholesalers of Colorado, the Smoke Free Alternative Trade Association, and JUUL Labs.

Some lobbyists and agencies represented multiple interests whose priorities appeared conflicted. We found 3 examples of lobbyists that represented both cannabis affiliates and health organizations. Gold Dome Access was paid $1,228,259 by the cannabis industry to lobby from FY2010–2021, and also received at least $11,795 from the American Heart Association FY2011–2013 and at least $23,887 from the Colorado Distiller’s Guild from 2016 to 2020. Young Public Affairs represented the cannabis business LivWell while also representing Anthem Blue Cross Blue Shield. Peak Government Affairs LTD concurrently lobbied on behalf of the Cannabis Chamber of Commerce, the American Heart Association, and the Colorado Licensed Beverage Association.

### Case study: Cannabis industry lobbying for onsite consumption establishments

The cannabis industry began formally advocating for licensing of onsite cannabis consumption establishments in 2017. Although a few “marijuana clubs” operated prior to 2019, these private organizations either grew cannabis for patrons, allowed them to bring their own, or traded cannabis for membership. In January 2017, SB063 proposed to create a new onsite consumption license for retail and medical dispensaries and an exemption from the Colorado Clean Indoor Air Act (CCIA) for smoking cannabis in a “Marijuana Club”. In the Senate Committee on Business, Labor, & Technology, the sponsor, Senator Marble (R), explicitly stated that the bill originated from the cannabis industry,

*“[This bill] has been 3 years in the making, put together by industry leaders and those involved in the growing and selling of marijuana and marijuana products. There has been huge discussion, stakeholder meetings, and this is the bill they’ve come up with.”*

Support for the bill came from cannabis businesses, consultants, and Pueblo County. Jason Warf, executive director and lobbyist for the Southern Colorado Cannabis Council compared secondhand cannabis smoke to incense and barbeque smoke and stated, “cannabis smoke is not harmful to the lungs.”

Public opposition to the bill came from health advocacy groups, hospital systems, professional associations, local governments, consultants, and Colorado Christian University, which all voiced concern over secondhand smoke exposure. RJ Ours, the Colorado Government Relations Director for the American Cancer Society Cancer Action Network (ACS CAN), indicated that health groups were excluded from stakeholder meetings, saying, “I think it’s unfortunate that ACS CAN and its partners didn’t have an opportunity to have some input in the language of the bill prior to its drafting.”

The bill survived less than three months before indefinite postponement by the Senate Committee on Business, Labor, and Technology in March 2017. On the same day, SB184, which would allow local governments to permit private membership cannabis clubs and clarify the constitutional definition of consumption that is conducted “openly and publicly” was heard in the same committee. Kevin Bommer of the Colorado Municipal League (CML) testified that the CML brought the bill to the legislative sponsors after it was initiated by the city of Trinidad. Renaissance Solutions, the Drug Policy Alliance, Terrapin Care Station, Denver relief Consulting, Schultz Public Affairs, and Pueblo County supported the bill while health groups including ACS CAN and the American Heart Association, hospital systems, and other local governments opposed. The House and Senate could not agree on amendments and the bill died in May.

Onsite cannabis consumption establishments were considered again in the 2018 session through HB1258. This bill proposed “Marijuana Accessory Consumption Establishments” for existing licensees and was supported by Dixie Brands, LivWell, Good Chemistry, Renaissance Solutions, Medicine Man, Native Roots, Gold Dome Access, and the Colorado Hotel and Lodging Association. It was opposed by ACS CAN, local governments, consultants, Colorado Association of Police Chiefs, and Colorado Christian University due to indoor air quality concerns related to indoor use of electronic smoking devices, which were excluded from the definition of “smoking” at the time. However, the Southern Colorado Cannabis Council and My420 tours opposed the bill because it could eliminate party bus cannabis tours and did not create true social consumption establishments. After passing the House and Senate, the bill was vetoed by Governor Hickenlooper amid concerns that it violated the Colorado Constitutional prohibition on “consumption that is conducted openly and publicly” (Hickenlooper, 2018). A parallel bill, SB211, was introduced in March 2018 by Senator Marble and would have allowed smoking in “consumption clubs” through an exemption to the Colorado Clean Indoor Air Act. The bill was again supported by Renaissance Solutions, Inc. and opposed by the City of Colorado Springs, Denver Health, Healthier Colorado, the American Heart Association, Smart Strategies, the Colorado Association of Chiefs of Police, ACS CAN, and the Colorado Association of Local Public Health Officials. It died in the Senate Committee on Business Labor and Technology in April. In contrasting HB1258 with SB211, David Wasserman, representing the Southern Colorado Cannabis Council, appealed to the history of collaboration between the cannabis industry and legislators,

*“My organization has had a 5-year stakeholder process on consumption, and we have worked with stakeholders from all sides during that time. … These clubs have worked with our organization and lawmakers for the past 5 years to create a license.”*

Governor Hickenlooper’s term ended in January 2019, and he was replaced by Colorado’s so-called “pot Governor,” (Frank, 2019) Jared Polis. HB1230, introduced on March 8, 2019, proposed state-licensed “Marijuana Hospitality Spaces” that would permit onsite consumption via smoking, vaping, and ingestion, if approved by local governments. It attracted support from cannabis businesses, trade associations, consultants, and advocacy organizations as well as local governments. Senator Marble again sponsored the bill, but unlike prior years, cannabis businesses from out-of-state, including Nuka Enterprises (New York) and Eaze Solutions (California) joined the usual proponents in support. Jason Warf of the Southern Colorado Cannabis Council stated during public testimony for the bill that,

*“Our organization in 2013 actually pushed it away and didn’t want anything to do with it. What happened between 2013 and 2014 is actually our licensees came to us and said we need to provide a place for safe consumption.”*

The bill was opposed by ACS CAN, the Group to Alleviate Smoking Pollution (GASP), The American Heart Association, professional associations, consultants, and other local governments. It also drew opposition from The Green Solution, a cannabis company, which opposed a state licensing system in favor of complete local control. The Colorado Brewer’s Guild lobbied to amend the bill when an amendment would have aligned business liability for impaired driving with that for bars serving alcohol and Anheuser-Busch, The Tavern League of Colorado, and Wine and Spirit Wholesalers of Colorado paid lobbyists to monitor the bill.

Each iteration of onsite cannabis consumption bills was supported by cannabis businesses (e.g., Terrapin Care Station, Renaissance Solutions) and opposed by health groups (e.g., ACS CAN, the American Heart Association). HB1230, exempting “Marijuana Hospitality Spaces” from the Colorado Clean Indoor Act was enacted on May 22, 2019, and signed by Governor Polis on May 29, 2019.

## Discussion

Our findings suggest that after recreational legalization the cannabis industry expanded its lobbying activities and used tactics comparable to those used by similar industries seeking to promote consumption. The dramatic increase in cannabis industry lobbying expenditures over time mirrored growth of the cannabis industry following recreational legalization in November 2012, which also coincided with an increase in cannabis consumption. Funding originating from out-of-state sources also increased over time, suggesting the development of a national network of cannabis affiliates with similar interests. Legislators, public health advocates, and community organizers should therefore expect industry resistance to cannabis control measures from local and national sources as well as proactive industry efforts to promote consumption and profits through policymaking channels.

We also found that cannabis lobbying lacked transparency. Colorado lobbyists characterized their clients ambiguously almost half of the time, meaning that cannabis affiliates could only be identified through lengthy investigation. These characterizations resulted in the appearance that many funders supported (or opposed) some proposed legislation, which may have created a false impression of a broad coalition. In reality these interests shared common owners, represented the same professional associations, and used the same lobbyists. We also found some evidence suggesting that public relations agencies may have hidden cannabis industry funding by paying salaried lobbyists on the behalf of funders without identifying them. To improve transparency, the Colorado Sunshine Law could be strengthened by a requirement in C.R.S. 24–6–301 §1.9 (XI) that lobbyists disclose their client’s identity as a cannabis business or any cannabis affiliation they hold under the “industry type” field (1962 & rev. 2020). To accomplish this, a revision of section 1 of the same statute may also be needed to eliminate the provision protecting clients from disclosure of “the names of any of its shareholders, investors, business partners, coalition partners, members, donors, or supporters, as applicable.” These changes would easily allow researchers and members of the public to identify cannabis clients as such using the CDOS website and facilitate improved legislative accountability.

Cannabis affiliates used lobbyists focused solely on cannabis as well as sharing lobbyists with other industries including tobacco, alcohol, pharmaceutical, and gaming. Like other industries, the cannabis industry is likely to work with these business interests to further their own profits. Using the same tactics employed by these industries, cannabis industry representatives self-reported lobbying positions opposing clean indoor air laws, health warnings for pregnant women, and potency restrictions, while supporting investment, onsite consumption, and access to medical cannabis in schools.

Cannabis industry funding peaked in 2019, which may be related to the change in state governor: Governor Hickenlooper (2011–2019) was moderate on cannabis, vetoing several pro-cannabis bills, while successor Governor Polis had voiced support for the cannabis industry (Polis, 2018) and was publicly supported by cannabis affiliates. The industry may have viewed his first year in office as an opportunity to pass pro-cannabis industry bills, including cannabis hospitality businesses, that had failed in previous years (Eason, 2019; Vendituoli, 2019).

In light of the sophisticated and well-financed influence campaign conducted by the cannabis industry, policymakers should push for stricter separation between the industry and the policymaking process. Frameworks designed to prevent undue influence from other commercial determinants of health including the alcohol, food, and tobacco industries can dampen industry influence by creating firewalls between corporations and policymakers. Example policies, including the guidelines for implementation of Article 5.3 of the Framework Convention on Tobacco Control (World Health Organization, 2008), the World Health Organization’s Framework for Engagement with Non-State Actors (World Health Organization, 2016), and the Office of Economic Co-operation and Development’s recommendations for preventing policy capture (OECD, 2017), could serve as starting points. These frameworks stand in opposition to the system of private interest institutionalism in Colorado which encourages the inclusion of all stakeholders and prompts regulators to make policies that synthesize stakeholder input. If formal mechanisms preventing cannabis industry influence in policy are not established, legislators should at least guarantee an equal voice to health advocates through balanced and accessible stake holding processes.

Our research has limitations. For public relations and law firms who represented multiple interests, expenditures that were not explicitly delineated as being from cannabis companies were not included in our analysis as the origin of funds could not be identified. For this reason, lobbying expenditures are likely undercounted. Second, the exact positions or intentions of cannabis industry affiliates on proposed bills could not necessarily be determined from the lobbying record; instead, where possible, we relied on legislative testimony. Next, the exclusion of salaries from lobbying agencies with ties to the cannabis industry to their employees may lead to an underestimation of the total influence exerted by cannabis interests. Finally, our description of lobbying expenditures did not include pro-bono industry lobbying activities conducted on behalf of cannabis affiliates. Future research might better characterize the legislative goals of the cannabis industry using additional review of campaign expenditures, legislative testimony, and using key informant interviews.

## Conclusion

Cannabis use is not necessarily harmless, and higher levels of consumption are associated with negative health outcomes. Our results suggest that an unintended effect of recreational cannabis legalization was an expansion of industry activities that can compromise public health, including advocacy for policies intended to increase cannabis use. Research on commercial determinants of health has found that tobacco, alcohol, and food interests have developed multiple tactics to encourage policy changes that encourage consumption, including hiring lobbyists, obscuring industry funding, and building alliances with related industries. The cannabis industry in Colorado began using all these strategies following recreational legalization, and alliances with related industries may have strengthened their coalition. The expansion of cannabis industry advocacy in Colorado led in at least one case to public health advocates being excluded from the development of policy, and ultimately resulted in the legalization of cannabis consumption establishments that are exempted from clean indoor air laws. Ensuring appropriate regulation of products that pose a risk to public health requires increased transparency to reveal relationships between cannabis affiliates, related industries, and policymakers, and providing an equal voice to health advocates.

## Figures and Tables

**Table 1:** Top 10 highest paid lobbyists/agencies by total payment (in inflation adjusted $) from cannabis affiliates from fiscal years 2010–2021.

Lobbyist/Agency	Client name	Client state	Total payment

Gold Dome Access	Medical Marijuana Industry Group	CO	1,209,518
	Dispensary Owners Coalition	CO	18,741
VS Strategies	Nuka Enterprises, LLC	NY	161,947
	Lightshade, LLC	CO	146,412
	Buddy Boy	CO	85,471
	Maggie’s Farm, LLC	CO	78,514
	ORCL, LLC	OR	64,079
	Pax	CA	64,077
	Northwest Holding Group	CO	26,921
	Willow Industries	CO	11,442
	Bio365	CA	7413
Sewald Hanfling Public Affairs	Dixie Brands	CO	237,165
	Medicine Man	CO	162,577
	Schwazze	CO	106,352
	MedPharm Holdings	CO	81,400
Young Public Affairs	Livwell	CO	543,596
Kristen Thomson	The Green Solution	CO	180,362
	Good Chemistry	CO	85,479
	Mindful	CO	47,610
	Gaia PBM, LLC	CO	42,011
	Medical Marijuana Industry Group	CO	39,663
	Cannabis Business Alliance	CO	29,700
	Dispensary Owners Coalition	CO	23,728
	Greenwerkz, LLC	CO	15,879
	Yofumo Technologies, Inc.	CO	13,673
	RFSCA LLC, dba RootsRX	CO	7968
Dutko Worldwide/Grayling	National Concessions Group, Inc.	CO	224,954
	Vicente Sederberg, LLC	CO	71,751
Furman Political Strategies	Renaissance Solutions	CO	143,937
	Eaze Solutions	CA	130,636
Sovine Consulting	Hoban Law Group	CO	114,314
	United Cannabis	CO	110,989
	Denver Relief Consulting	CO	16,710
	Cannabis Consumer Coalition	CO	11,032
Shawn Coleman	Renaissance Solutions	CO	123,396
	NSG, LLC	CO	21,311
	Skinny Pineapple, Inc.	CO	20,651
	The Genetic Locker	CO	16,335
	BBM Enterprises	CO	9579
	AER Investments, LLC	CO	9200
	Vicente Sederberg, LLC	CO	8187
	Colorado Cannabis Tours	CO	5347
	Pioneer Industries	CO	5347
	Colorado Healing	CO	4569
Margaret Mary O’keefe	Cannabis Business Alliance	CO	153,898
	Colorado Cannabis Manufacturers Association	CO	59,974

## Data Availability

The data supporting the conclusions of this article are publicly available at https://www.sos.state.co.us/ and https://data.colorado.gov/.
